# Missed PrEP opportunities and vulnerability to HIV during rifampicin-resistant tuberculosis treatment in South Africa

**DOI:** 10.3389/fpubh.2026.1797716

**Published:** 2026-03-23

**Authors:** Adam Leonard, Kelly Lowensen, Elizabeth Di Giacomo, Brenice Duroseau, Ntombasekhaya Mlandu, Yakub Kadernani, Ricardo Arcêncio, Chakra Budhathoki, Denise Evans, Norbert Ndjeka, Jason E. Farley

**Affiliations:** 1 Center for Infectious Disease and Nursing Innovation (CIDNI), Johns Hopkins School of Nursing, Baltimore, MD, United States; 2 BringBPaL2Me (BB2) Trial, Gqeberha, South Africa; 3 College of Nursing at Ribeirão Preto, University of São Paulo, Ribeirão Preto, Brazil; 4 Health Economics and Epidemiology Research Office, Wits Health Consortium, University of the Witwatersrand, Johannesburg, South Africa; 5 Tuberculosis Control and Management, National Department of Health, Pretoria, South Africa

**Keywords:** HIV prevention, pre-exposure prophylaxis, rifampicin-resistant tuberculosis, sexual health, tuberculosis

## Abstract

**Background:**

Adults receiving rifampicin-resistant TB (RR-TB) treatment have prolonged engagement with the health system, yet healthcare professionals frequently miss opportunities to promote HIV prevention—particularly pre-exposure prophylaxis (PrEP)—in high-burden settings. We examined HIV vulnerability, PrEP awareness and uptake, and HIV and sexually transmitted infection (STI) incidence among HIV-negative adults receiving RR-TB treatment.

**Methods:**

We conducted an exploratory analysis within the BringBPaL2Me (BB2) Trial across two South African provinces. HIV-negative adults who had a BB2 study outcome within the first 24 months of the trial were included. Sexual risk indicators, HIV testing, and PrEP outcomes were assessed longitudinally. HIV and STI incidence rates were calculated per 100 person-years with exact Poisson 95% confidence intervals.

**Results:**

Among 288 participants (mean age 38.0 years; 72.9% male), HIV vulnerability was prevalent, with 45.8% reporting condomless sex and 5.9% empirically treated for an STI at enrollment. Forty-two percent were aware of PrEP; 60.8% were offered PrEP, yet only 10.1% initiated PrEP. Over 110.7 person-years of follow-up, six incident HIV infections occurred (5.42 per 100 person-years; 95% CI: 1.99–11.80) and empiric treatment for an STI resulted in an STI incidence of 9.66 per 100 person-years (95% CI: 4.99–16.88).

**Conclusion:**

HIV-negative adults receiving RR-TB care experience substantial and ongoing HIV risk, yet uptake of biomedical prevention remains limited. Strengthening integration of HIV prevention within TB services represents a critical opportunity to advance sexual health and reduce HIV incidence in high-risk, underserved populations.

## Introduction

In South Africa, the intersection of tuberculosis (TB) and HIV continues to shape the national health landscape. HIV remains the leading risk factor for developing TB, accounting for approximately 55% of TB cases and 69% of TB-related deaths over the past three decades (1). In 2023, an estimated 54% of individuals diagnosed with TB were living with HIV or AIDS (PLWHA) (2). While widespread antiretroviral therapy (ART) scale-up has contributed to declining TB incidence among PLWHA (1), little attention has been paid to preventing HIV.

This oversight is particularly significant in the context of rifampicin-resistant (RR-TB) treatment, which necessitates a continuum of care within the healthcare system over a six- to nine-month treatment period and extended post-treatment follow-up (3). Despite this prolonged and structured engagement with the health system and patient-centered adherence support required, HIV prevention services, particularly pre-exposure prophylaxis (PrEP), are not routinely integrated into RR-TB care pathways, resulting in missed opportunities to address ongoing sexual health needs among HIV-negative patients. In this context, we conceptualize missed opportunities for PrEP provision as clinical encounters during RR-TB care in which HIV-negative patients with ongoing HIV vulnerability engage with the health system but are neither assessed for PrEP eligibility, offered PrEP, nor initiated on PrEP despite potential indication. RR-TB care, unlike drug-susceptible TB, involves repeated visits over a longer treatment period and offers a unique programmatic opportunity to integrate sexual health and HIV prevention services for patients who may remain at risk throughout care.

Although TB services have appropriately focused on optimizing HIV diagnosis and viral suppression among PLWH (4, 5), little attention has been paid to preventing HIV among the substantial proportion of HIV-negative RR-TB patients (6). HIV increases the risk of complications and unfavorable outcomes for RR-TB (mainly extensively drug-resistant disease and death) and evidence consistently demonstrates ongoing HIV vulnerability among people receiving TB care, via report of condomless sex, multiple partnerships, and substance use that may exacerbate sexual risk (7–12). Preventing HIV acquisition in this population has the potential to improve TB treatment outcomes, lower mortality, and decrease TB recurrence (13–15). These benefits extend beyond individual-level outcomes to reduce population-level transmission of both drug-susceptible and drug-resistant TB (1, 16–19).

HIV PrEP is a highly efficacious and safe biomedical prevention strategy (20), with modeling studies in South Africa suggesting that 21–30% of new HIV infections could be averted with sufficient scale-up (21, 22). Despite recent expansion of PrEP availability, uptake remains uneven, and early discontinuation limits population-level impact (23). Notably, HIV incidence during RR-TB treatment has not been quantified in South Africa, limiting understanding of the magnitude and timing of HIV acquisition risk during TB care. Existing PrEP implementation efforts in South Africa have largely focused on HIV and reproductive health services and priority populations, with limited attention to TB and RR-TB treatment settings as delivery platforms. Implementation research in South Africa has demonstrated that differentiated and integrated PrEP delivery models can improve feasibility, acceptability, and reach across diverse populations and service settings (24–29). However, PrEP initiation continues to rely heavily on provider-initiated counseling and patients’ perceived risk–benefit assessments, processes that are shaped by interpersonal dynamics, clinic workflows, stigma, and broader structural conditions (30–32). These dynamics have been insufficiently examined within TB and RR-TB care contexts, leaving a gap in our understanding of how best to offer and scale PrEP in TB programs.

To our knowledge, this is the first study to examine PrEP integration into RR-TB treatment programs and to characterize HIV vulnerabilities, including STI burden and HIV incidence, among HIV-negative individuals receiving RR-TB care in South Africa. Addressing this gap is particularly salient given the World Health Organization’s recommended patient-centered approach and integration of HIV and TB care.

## Materials and methods

### Study design

This exploratory analysis includes HIV-negative participants enrolled in the ongoing BringBPaL2Me Trial (BB2), a NIAID-funded, multi-site, cluster-randomized non-inferiority trial comparing nurse-led versus physician-led RR-TB treatment (R01AI177135, https://www.clinicaltrials.gov/study/NCT05671718). Participants diagnosed with RR-TB were recruited from 76 public-sector primary care clinic clusters across three municipalities in KwaZulu-Natal and two in the Eastern Cape, South Africa. Recruitment was facilitated through referrals from clinic TB focal nurses to study research assistants. The study arms received the same treatment: six months of Bedaquiline, Pretominanid, Linezolid, and Levofloxacin, administered by either a physician or a nurse. Clinician visits occurred at baseline, week two, and then every four weeks until treatment completion at month six (or month nine in extended treatment cases). Patients could also be seen for unscheduled visits as needed for medication-related adverse events, worsening symptoms, or general health complaints.

### Study population

The sample included adults who consented to the parent trial, were eligible for primary care RR-TB management, and had a documented RR-TB treatment outcome within the first 24 months of the study period (September 2023–August 2025). Participants were included in the present analysis if they were documented HIV-negative at baseline. Exclusion criteria for the parent trial include pregnancy, severe anemia, ALT > 120 U/L, liver disease, prolonged QTc (>470 ms), tachycardia (>140 beats per minute), symptoms of extrathoracic TB, illness severity necessitating hospital-based care, or concurrent enrollment in another clinical trial affecting RR-TB treatment.

### Data collection and measures

Demographic variables included age, sex, race, education level, relationship status, and living arrangement. Data were collected by trained research assistants fluent in English and in locally relevant South African languages (including Afrikaans, isiXhosa, and isiZulu, depending on the region). Data were entered directly into a REDCap secure electronic database using tablet-based devices. Data quality assurance procedures, including routine review for completeness and consistency, were conducted by the study coordinator, project manager, principal investigator, and affiliated doctoral trainees.

HIV testing and prevention data were collected at baseline, months three and six, monthly for those who had clinically indicated treatment extension (up to nine months), and at a final six-month post-treatment completion visit. The PrEP continuum was operationalized as baseline PrEP awareness, PrEP offer, and PrEP acceptance at any study visit. Per study protocol, PrEP awareness was assessed at baseline; participants who were unaware received education and clinicians were instructed to offered PrEP, while those who were already aware were offered PrEP. PrEP acceptance was defined as a participant response of “yes” to accepting PrEP and confirmed by documentation of tenofovir/emtricitabine (TDF/FTC) on the participant’s medication list.

HIV incidence was defined as seroconversion during follow-up and expressed per 100 person-years. Person-time accrued from the date of study enrollment to the first documented HIV-positive test or the last documented HIV-negative test during treatment follow-up, whichever occurred first. Participants without a documented HIV test at the final treatment visit but with a negative test at the six-month post-treatment visit were classified as HIV-negative at treatment completion. Participants without any documented post-baseline HIV testing did not contribute person-time to incidence estimates; therefore, HIV incidence reflects observed follow-up time among participants with documented HIV testing.

STI prevalence was defined using a syndromic and treatment-based algorithm consistent with standard practice in South Africa. This algorithm required both (1) documentation of treatment with one or more STI-related medications (including azithromycin, benzylpenicillin, ceftriaxone, doxycycline, or metronidazole) and (2) documentation of one or more STI symptoms (genital discharge, rash, lesion, itching, pain, or painful urination) or a reactive rapid plasma reagin (RPR) test for syphilis. Person-time for incident STI accrued from the date of study enrollment to the date of first documented STI treatment or to the date of the last documented clinic visit during the treatment period for participants without an incident STI; participants with a prevalent STI at baseline were excluded from STI person-time calculations.

### Statistical analysis

Descriptive statistics summarized participant characteristics, HIV vulnerability indicators, and PrEP continuum outcomes. Follow-up HIV testing during the study period was summarized cumulatively across scheduled follow up visits, with documented testing defined by the presence of a recorded HIV test result; participants were classified as having zero, one, or two documented follow-up tests. Incidence rates for HIV and sexually transmitted infections were calculated per 100 person-years, with exact 95% confidence intervals estimated under a Poisson distribution given the small number of observed events. Baseline PrEP awareness (aware vs. not aware) was examined in relation to PrEP engagement using categorical analyses aligned with the PrEP cascade. We first conducted a global Pearson chi-square test to compare the distribution of a three-category PrEP outcome (never offered PrEP, offered PrEP but never initiated, initiated PrEP) across baseline awareness status. Given evidence of a global association, we performed chi-square tests to localize the association along the cascade. Specifically, we compared (1) ever being offered PrEP versus never being offered PrEP, and (2) PrEP initiation versus non-initiation among those offered PrEP.

We conducted descriptive case-ascertainment sensitivity analyses to evaluate potential bias from incomplete follow-up HIV testing. Holding observed person-time constant (i.e., restricting the denominator to participants with ≥1 post-baseline HIV test), we recalculated HIV incidence under scenarios assuming 1 or 2 additional, unobserved seroconversions among participants without documented follow-up testing. These scenarios do not impute follow-up time for untested participants and are intended to illustrate the sensitivity of the incidence estimate to plausible under-ascertainment of events.

### Sample size justification

Sample size for the parent BB2 Trial was determined by its ongoing primary non-inferiority objectives related to RR-TB treatment delivery and was not based on detecting HIV incidence among HIV-negative participants. The present analysis of HIV seroconversion was based on available and relevant data; therefore, it is secondary and exploratory. No formal sample size or power calculation was conducted for this outcome.

### Ethics statement

All data elements were collected under the approved BB2 protocol, with ethical oversight provided by Johns Hopkins University, the University of the Witwatersrand, and the relevant provincial research authorities (Clinical Trials ID NCT05671718).

## Results

A total of 288 HIV-negative adults from 76 public-sector clinic clusters in the KwaZulu-Natal and Eastern Cape provinces were included in this study, representing all HIV-negative participants enrolled in the larger BB2 study who had a study outcome by the end of the follow-up period. Participants had a mean age of 38.0 years (SD 13.9); 72.9% were male, 75.0% identified as Black South African, and 72.6% lived in informal settlements. Additional demographic characteristics by PrEP use status are presented in Table 1.

**Table 1:** Demographic and HIV vulnerability profile of the sample by PrEP use**.

Variable	PrEP non-users^a^ (*n* = 259) *n* (%)^b^ or mean (SD)	PrEP users^a^ (*n* = 29) *n* (%)^b^ or mean (SD)	Total (*n* = 288) *n* (%) or mean (SD)
Age	38.3 (14.2)	35.4 (10.1)	38.0 (13.9)
Sex			
Male	188 (72.6%)	22 (75.9%)	210 (72.9%)
Female	71 (27.4%)	7 (24.1%)	78 (27.1%)
Race			
Black South African	190 (73.4%)	26 (89.7%)	216 (75.0%)
Mixed Racial Background	62 (23.9%)	3 (10.3%)	65 (22.6%)
Other	7 (2.7%)	0 (0.0%)	7 (2.4%)
Marital status			
Single	222 (85.7%)	25 (86.2%)	247 (85.8%)
Married	27 (10.4%)	1 (3.4%)	28 (9.7%)
Separated/Divorced/Widowed	10 (3.9%)	3 (10.3%)	13 (4.5%)
Education			
Primary or less	147 (56.8%)	12 (41.4%)	159 (55.2%)
Secondary	104 (40.2%)	16 (55.2%)	120 (41.7%)
Technical/College	8 (3.1%)	1 (3.4%)	9 (3.1%)
Lives in informal settlement			
Yes	184 (71.0%)	25 (86.2%)	209 (72.6%)
No	75 (29.0%)	4 (13.8%)	79 (27.4%)
Province			
KwaZulu-Natal	62 (23.9%)	12 (41.4%)	74 (25.7%)
Eastern Cape	197 (76.1%)	17 (58.6%)	214 (74.3%)
HIV Vulnerabilities			
Reported condomless sex at least at one visit			
Yes	117 (45.2%)	15 (51.7%)	132 (45.8%)
No	142 (54.8%)	14 (48.3%)	156 (54.2%)
Treated for a sexually transmitted infection^c^			
No STI	245 (94.6%)	26 (89.7%)	271 (94.1%)
Prevalent STI (baseline)	4 (1.5%)	1 (3.4%)	5 (1.7%)
Incident STI (follow-up)	10 (3.9%)	2 (6.9%)	12 (4.2%)
Any sexual risk behavior^d^			
Yes	121 (46.7%)	18 (62.1%)	139 (48.3%)
No	138 (53.3%)	11 (37.9%)	149 (51.7%)

PrEP, pre-exposure prophylaxis; STI, sexually transmitted infection.

^a^PrEP use was defined as initiation of PrEP at any study visit (baseline, month 3, or month 6).

^b^Percentages are calculated within PrEP use categories.

^c^Prevalent STI indicates infection at baseline; incident STI indicates infection during follow-up.

^d^Any sexual risk behavior was defined as reporting condomless sex at any visit and/or treatment for an STI during follow-up.

**Figure 1:**
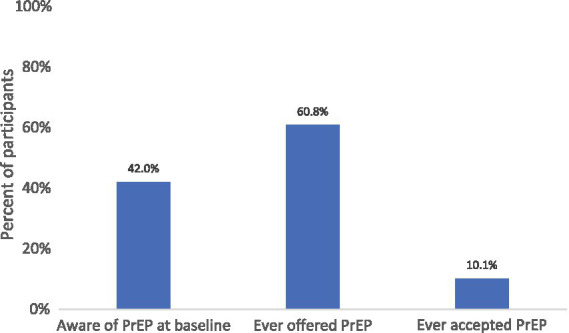
PrEP awareness, offering, and uptake among study participants (*n* = 288). PrEP awareness at baseline, PrEP offering, and PrEP uptake among study participants. Percentages are calculated using the full study population (*N* = 288). PrEP offering and uptake are recorded at any study visit (baseline, month-3, or month-6).

**Figure 2:**
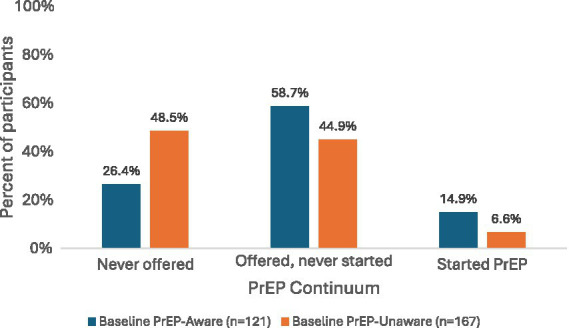
PrEP offering and uptake by baseline PrEP awareness. Bars represent the proportion of participants who were never offered PrEP, offered but never started PrEP, and who started PrEP, stratified by baseline PrEP awareness (aware at baseline, *n* = 121; unaware at baseline, *n* = 167). Percentages are calculated within baseline awareness groups. PrEP offering and uptake were recorded at any study visit (baseline, month-3, or month-6).

Indicators of ongoing HIV vulnerability were prevalent in the study population. Overall, 132 participants (45.8%) reported condomless sex at one or more study visits, 12 participants (4.2%) were treated for an incident sexually transmitted infection during follow-up, and nearly half of participants (48.3%) met criteria for any sexual risk behavior, defined as condomless sex and/or STI treatment (Table 1). At enrollment, 121 participants (42.0%) reported awareness of PrEP. Overall, 175 participants (60.8%) were offered PrEP during follow-up, and 29 (10.1%) initiated PrEP at any study visit. Baseline PrEP awareness, subsequent offering, and uptake across the full study population are shown in Figure 1. When stratified by baseline PrEP awareness, the distribution of PrEP offering and uptake differed between participants who were aware of PrEP at enrollment and those who were unaware (Figure 2). Among participants unaware of PrEP at baseline, 48.5% were never offered PrEP, 44.9% were offered but did not initiate, and 6.6% initiated PrEP; among those aware of PrEP at baseline, 26.4% were never offered PrEP, 58.7% were offered but did not initiate, and 14.9% initiated PrEP. Because all HIV-negative participants were considered potentially eligible for PrEP under the study protocol, the category of “never offered PrEP” reflects the absence of a documented clinician offer during follow-up. In contrast, the category of “offered but never started” reflects patient-level decision-making after a documented PrEP offer.

Baseline PrEP awareness was associated with differences in the distribution of PrEP engagement categories (χ^2^ (2) = 16.1, *p* < 0.001). Participants who were aware of PrEP at baseline were less likely to have never been offered PrEP and more likely to have been offered or initiated PrEP compared with those who were unaware. Baseline awareness was associated with ever being offered PrEP (χ^2^ (1) = 14.3, p < 0.001). In contrast, among participants who were offered PrEP, baseline awareness was not associated with subsequent PrEP initiation (χ^2^ (1) = 1.75, *p* = 0.19).

Over 110.7 person-years of HIV follow-up, six incident HIV infections were observed (four male, two female), yielding an incidence rate of 5.42 per 100 person-years (95% CI: 1.99–11.80). Five of the six seroconversions (83%) were detected at the Month 3 visit, with one additional infection identified at extended-treatment completion (month nine). No seroconversions occurred while participants were receiving PrEP as prescribed for HIV prevention. Five of the six individuals who seroconverted had never initiated PrEP. The remaining individual was unaware of PrEP at baseline and was never offered PrEP; this participant was later prescribed tenofovir/emtricitabine (TDF/FTC) for treatment of hepatitis B but discontinued TDF/FTC prior to HIV diagnosis. Thus, seroconversion did not occur during active tenofovir-based prevention exposure. For analytic purposes, this participant was classified as a non–PrEP user because TDF/FTC was not initiated for HIV prevention and was not being taken at the time of acquisition.

Over 124.2 person-years, 12 incident sexually transmitted infections (eight male and four female) were identified, yielding an incidence rate of 9.66 per 100 person-years (95% CI: 4.99–16.88).

Follow-up HIV testing was documented for 85.1% of participants, including 67.4% with documented testing at two visits and 17.7% with testing at a single follow-up visit. The proportion of participants without documented follow-up HIV testing (14.9%) varied across provinces and metropolitan or municipal areas, as shown in Table 2. Incident HIV infections occurred across multiple clinics rather than being concentrated at a single site. To assess the potential impact of incomplete follow-up testing, we conducted case-ascertainment sensitivity analyses holding observed person-time constant (110.7 PY). Assuming one additional unobserved seroconversion among participants without documented follow-up testing increased the incidence to 6.32 per 100 person-years; assuming two additional unobserved seroconversions increased the incidence to 7.23 per 100 person-years.

**Table 2:** HIV follow-up testing outcomes (months-3 and/or 6-), by province and metropolitan/municipal area.

HIV follow-up test	KwaZulu-Natal province			Eastern Cape province	
	eThekwini metropolitan (Durban) *n* = 66	Msunduzi municipality (Pietermaritzburg) *n* = 5	Ray Nkonyeni municipality (Pt Shepstone) *n* = 3	Buffalo City Metropolitan (East London) *n* = 84	Nelson Mandela Bay Metropolitan (Gqeberha) *n* = 130
HIV test negative	56 (84.9%)	4 (80.0%)	3 (100%)	73 (86.9%)	103 (79.2%)
HIV test positive	1 (1.5%)	1 (20.0%)	0 (0.0%)	3 (3.6%)	1 (0.8%)
No follow-up HIV test	9 (13.6%)	0 (0.0%)	0 (0.0%)	8 (9.5%)	26 (20.0%)

Values are counts of participants with column percentages shown in parentheses. Percentages are calculated using all participants enrolled in each metropolitan or municipal area as the denominator. “No follow-up HIV test” indicates participants without a documented HIV test after baseline during the study follow-up period (month-3 and/or month-6). HIV-positive results during follow-up were classified as incident infections when a prior negative test was documented at baseline. Incident HIV infections observed during follow-up were distributed across multiple clinics rather than concentrated at a single site; in Buffalo City Metropolitan Municipality (East London), the three incident cases occurred at different clinics.

## Discussion

This study highlights substantial unmet sexual health and HIV prevention needs among HIV-negative adults receiving RR-TB treatment in South Africa. This population remains absent from HIV prevention research and programming, and this is the first observational study detailing programmatic integration of PrEP services within an RR-TB treatment program. Despite monthly patient engagement for six to nine months, followed by quarterly engagement for up to 12 months after treatment within the RR-TB program creating a perfect opportunity for HIV prevention, PrEP uptake was low, even in the context of documented HIV vulnerabilities, ongoing sexual risk, and a high incidence rate. These findings underscore a persistent gap between biomedical prevention availability and meaningful access, reflecting not only interpersonal and structural factors but also clinical barriers within TB-focused care settings, including competing visit priorities and provider workflow constraints that may limit integration of HIV prevention services (33). Although PrEP counseling and offering were included in clinical protocols, low PrEP uptake suggests challenges in translating these protocols into routine practice, likely reflecting constraints related to clinic workflows, provider practices, and integration of HIV prevention within TB-focused care.

From an implementation perspective, the observed gaps across HIV vulnerability, follow-up HIV screening, PrEP offering, and PrEP uptake point to multilevel barriers operating within RR-TB care. At the provider level, PrEP delivery in TB settings may be constrained by competing clinical priorities, limited time for sexual health counseling during visits focused on complex RR-TB management, and discomfort or lack of training in discussing sexual behavior with patients who are not traditionally perceived as “high risk.” Even within a protocolized research environment, these factors may contribute to missed opportunities for PrEP offering, particularly among patients who are unaware of PrEP at baseline and therefore rely entirely on provider-initiated counseling. The lack of a specific PrEP counseling job aid may have been one contributing factor to low rates of provider PrEP counseling, despite its inclusion in study protocol materials.

At the clinic level, workflows in TB services are typically organized around medication delivery, symptom monitoring, and management of treatment-related adverse events. Sexual health discussions and HIV prevention counseling may be deprioritized in the absence of explicit integration into visit structures, standardized screening tools, or performance indicators tied to prevention outcomes. This is particularly relevant in RR-TB care, where visit time is often dominated by adherence monitoring and management of adverse drug reactions, leaving limited space for proactive prevention counseling unless it is deliberately embedded within routine care processes.

Structural and policy-level factors further shape these implementation gaps. HIV prevention and TB care are frequently governed by parallel guidelines, reporting systems, and funding streams, which can reinforce service silos even in settings where patients experience overlapping risks. In the absence of explicit guidance or accountability mechanisms for PrEP delivery within TB programs, prevention efforts may depend on individual provider initiative rather than system-level design. Together, these findings suggest that improving PrEP uptake in RR-TB settings will require interventions that address provider training, clinic workflow redesign, and policy alignment, rather than solely focusing on patient-level demand creation.

The substantial proportion of participants who were offered PrEP but did not initiate highlights implementation challenges related to pill burden, competing treatment demands during intensive TB therapy, and the framing of HIV prevention as ancillary to RR-TB care. In this context, the recent approval of long-acting injectable PrEP options in South Africa presents a promising opportunity to address these barriers by reducing daily adherence demands and better aligning HIV prevention with the realities of RR-TB treatment.

Although specific reasons for non-initiation were not systematically captured, several plausible multilevel factors may have contributed. At the patient level, individuals undergoing RR-TB treatment face substantial pill burden, medication-related adverse effects, and competing health priorities, which may reduce perceived capacity to initiate an additional preventive medication. Perceived HIV risk may also have been low among some participants despite documented vulnerability indicators. At the interpersonal level, stigma related to HIV prevention, concerns about confidentiality, and limited opportunity for shared decision-making during clinically intensive TB visits may have influenced uptake.

Observed HIV incidence during RR-TB treatment exceeded estimates for the general South African adult population, reinforcing the concept of TB care as a critical but underutilized point of intervention for HIV prevention (34). Modeling studies demonstrate that TB-affected households harbor disproportionately high HIV risk, with substantial preventable HIV acquisition if PrEP were systematically offered within TB platforms (12). Interpretation of HIV incidence estimates in this study requires careful contextualization. Direct comparisons to population-level estimates are limited by differences in sampling, follow-up duration, and risk composition. In this cohort, five of six seroconversions (83%) were detected by the Month 3 visit, with one additional infection identified later in treatment. Thus, incident HIV acquisition occurred predominantly early in the treatment course rather than clustering in the later recovery phase. While sexual activity and perceived risk may shift as patients clinically improve, these data suggest that vulnerability is present from treatment initiation. The occurrence of early seroconversions underscores the importance of proactive HIV prevention counseling and PrEP offering at or near RR-TB treatment start, rather than deferring prevention discussions to later visits (35).

One seroconversion occurred in a participant who had been prescribed tenofovir/emtricitabine (TDF/FTC) for hepatitis B treatment but discontinued therapy prior to HIV diagnosis. Importantly, HIV acquisition did not occur during active tenofovir-based exposure. This case does not suggest biologic failure of PrEP; rather, it highlights implementation challenges within RR-TB care. Participants undergoing intensive TB treatment may experience substantial pill burden, competing clinical priorities, and medication fatigue, all of which can affect persistence with additional therapies. In this context, tenofovir prescribed for hepatitis B was not framed or monitored as HIV prevention, representing a missed opportunity to align existing tenofovir-based treatment with prevention counseling and adherence support. This case underscores the need to integrate HIV risk assessment and prevention framing into routine medication management within TB services, including consideration of how tenofovir-containing regimens prescribed for other indications might be leveraged as part of a comprehensive HIV prevention strategy.

The timing of HIV acquisition during RR-TB treatment is particularly salient. RR-TB care involves prolonged engagement with the health system, yet HIV prevention is often addressed episodically or deferred once initial HIV-negative status is documented. The occurrence of seroconversions during follow-up highlights the dynamic nature of HIV risk and the limitations of one-time risk assessment at treatment initiation. These findings support the need for repeated, longitudinal prevention counseling that aligns with patients’ evolving circumstances throughout treatment.

From a programmatic perspective, estimating HIV incidence within TB care trajectories provides actionable information that is often missing from routine surveillance. While TB programs routinely monitor treatment outcomes and adverse events, HIV acquisition among HIV-negative patients receiving TB care is rarely tracked. Incorporating prevention-relevant indicators into TB monitoring systems could help identify high-risk periods, inform service integration strategies, and support more responsive, patient-centered prevention efforts. The concurrent burden of symptomatic STIs further signals unmet sexual health needs and highlights the interconnected biological and social pathways through which HIV risk is produced and sustained (36). Global Burden of Disease estimates indicate that South Africa has among the highest age-standardized STI incidence rates globally, reflecting a high background burden of sexually transmitted infections (37). In this context, the observed incidence of symptomatic STIs during RR-TB treatment likely represents an underestimation of true STI burden, given reliance on syndromic management and the high prevalence of asymptomatic infection. Taken together, these findings underscore that HIV and STI risk during RR-TB treatment cannot be attributed solely to individual behavior but instead reflect the interaction of structural constraints, stigmatized service environments, and limited integration of sexual health services within TB programs (12).

Integrating PrEP into decentralized, nurse-led TB services represents an opportunity to leverage existing system strengths to advance sexual health and wellness (38). TB clinics offer routine, longitudinal contact, trusted provider relationships, and established medication-delivery platforms that could support integrated psychosocial and biomedical prevention approaches (33). Embedding PrEP within TB care aligns with differentiated service delivery models and reflects principles of cultural humility by meeting patients where they already receive care, rather than requiring navigation of fragmented or stigmatized services (39). Nurse-led, decentralized TB services are particularly well positioned to support integrated sexual health care (40). Nurses often develop sustained therapeutic relationships with patients over the course of RR-TB treatment, creating opportunities for trust-building and patient-centered counseling that can facilitate sensitive discussions about sexual health. Task-shifting PrEP counseling and initiation to nursing professionals within TB programs aligns with broader differentiated service delivery models and may enhance scalability, particularly in resource-constrained settings.

Conceptualizing TB clinics as platforms for sexual health and HIV prevention reframes integration not as an added burden, but as an extension of existing system strengths. RR-TB care is characterized by longitudinal engagement, frequent patient–provider contact, and established medication delivery systems, features that are well aligned with the requirements of PrEP initiation and follow-up. In contrast to episodic or referral-based prevention models, TB services offer repeated opportunities to assess HIV risk, provide tailored counseling, and revisit prevention decisions over time, which may be particularly important for patients navigating complex treatment regimens and evolving perceptions of risk.

For HIV-negative individuals receiving RR-TB treatment, TB clinics may also represent comparatively lower-stigma environments for engaging in HIV prevention than HIV-specific services. Patients entering TB care do not necessarily self-identify as members of populations traditionally targeted by PrEP programs and may therefore be less likely to seek out prevention services independently. Embedding PrEP within TB services has the potential to reduce stigma by normalizing HIV prevention as a routine component of comprehensive care rather than a marker of perceived risk or identity. Viewed through an asset-based and ecological lens, integrating HIV prevention into RR-TB care leverages existing clinical infrastructure to address intersecting health needs shaped by structural vulnerability. Rather than requiring patients to navigate fragmented systems, this approach brings prevention to where care is already occurring, supporting autonomy, equity, and holistic sexual health in line with global calls to reframe sexual health as a core component of well-being.

This study should be interpreted considering several limitations. First, the analysis was descriptive and embedded within a parent clinical trial that was not designed to evaluate HIV prevention outcomes, limiting causal inference and precluding assessment of the effectiveness of PrEP integration into RR-TB care. This analysis was not prospectively powered to estimate HIV incidence for comparison to population-level estimates. We report achievable precision based on accrued person-time. With 6 seroconversions over 110.7 person-years, the exact Poisson 95% confidence interval (1.99–11.80 per 100 person-years) is wide, reflecting substantial statistical uncertainty. The point estimate (5.42 per 100 person-years) should thus be interpreted as evidence that HIV acquisition occurred during RR-TB care rather than as a stable population-level rate suitable for formal comparison with national surveillance estimates. These findings are exploratory and hypothesis-generating.

Second, Follow-up time for HIV incidence was limited to the duration of RR-TB treatment and associated follow-up visits, which may underestimate longer-term HIV risk following treatment completion. Because HIV person-time accrued only among participants with documented post-baseline HIV testing, incidence estimates may be influenced by differential loss to follow-up or incomplete testing during the study period. To assess the potential impact of incomplete follow-up testing, we conducted case-ascertainment sensitivity analyses holding observed person-time constant; these analyses demonstrated that, given the small number of observed events, the incidence estimate is sensitive to modest changes in case ascertainment. These scenarios did not impute follow-up time or outcomes for untested participants and should be interpreted as illustrative rather than corrective.

Third, STI prevalence was identified using syndromic management and treatment documentation rather than laboratory-confirmed diagnoses, which likely underestimates the true burden of asymptomatic infections and may misclassify some cases. Fourth, PrEP acceptance was defined based on documented agreement and confirmation of medication prescription, but data on PrEP adherence, persistence, and discontinuation were not analyzed. Measures of sexual risk (e.g., condomless sex) were based on self-report and may be subject to social desirability bias in stigmatized care contexts. Finally, although the study included participants from multiple clinics across two provinces, the findings may not be generalizable to all TB treatment settings, particularly those with different service-delivery models or HIV-prevention infrastructure.

These limitations point to important implications for future research and program design. Reliance on syndromic STI management, while consistent with routine care in South Africa, likely underestimates the burden of asymptomatic infection and highlights missed opportunities for more comprehensive sexual health screening within TB services. Similarly, the restriction of HIV person-time to participants with documented follow-up testing underscores the need for more systematic, protocolized HIV re-testing during RR-TB treatment. These limitations reflect the realities of current TB care and surveillance systems. They suggest that integrating HIV prevention into TB programs will require not only expanded service delivery but also strengthened data systems capable of capturing longitudinal sexual health outcomes. Embedding standardized HIV risk reassessment, repeat testing, and prevention indicators into TB care could improve both individual patient outcomes and program-level accountability.

Taken together, these considerations reinforce the value of implementation-focused research that addresses how sexual health services can be sustainably integrated into TB care under real-world conditions, rather than relying on parallel or referral-based prevention models that may exacerbate fragmentation and inequities. These findings support calls to reframe sexual health as a human right and a core component of comprehensive TB care, rather than an ancillary or optional service. Explicit inclusion of HIV prevention, including PrEP, within national TB and RR-TB guidelines could help normalize sexual health discussions, reduce stigma, and promote equity by ensuring that populations historically overlooked in HIV prevention efforts are reached. This approach aligns with the End TB Strategy, one of whose pillars emphasizes patient-centered care and equity.

Future research should focus on implementation strategies that address multilevel determinants of PrEP uptake within TB settings, including provider training, clinic workflows, patient-centered counseling, and structural supports. Hybrid effectiveness–implementation studies are needed to evaluate not only PrEP uptake and persistence but also acceptability, feasibility, and sustainability within real-world TB programs. Such work would directly contribute to global goals of ending both the HIV and TB epidemics while advancing a holistic, asset-based vision of sexual health and wellness across the lifespan.

## Data Availability

The raw data supporting the conclusions of this article will be made available by the authors, without undue reservation.
